# Collaborative care for child and youth mental health problems in a middle-income country: study protocol for a randomized controlled trial training general practitioners

**DOI:** 10.1186/s13063-019-3467-4

**Published:** 2019-07-08

**Authors:** Vandad Sharifi, Zahra Shahrivar, Hadi Zarafshan, Sheida Beiky Ashkezary, Elizabeth Stuart, Ramin Mojtabai, Lawrence Wissow

**Affiliations:** 1 Department of Psychiatry, Tehran University of Medical Sciences, Tehran, Iran; 2 Psychiatry and Psychology Research Center, Tehran University of Medical Sciences, Tehran, Iran; 3 Department of Mental Health, Bloomberg School of Public Health, Johns Hopkins University, Baltimore, MD, USA; 4 Division of Child and Adolescent Psychiatry, School of Medicine, University of Washington, 4800 Sand Point Way NE, M/S OA.5.154, PO Box 5371, Seattle, WA 98145-5005 USA

**Keywords:** Children, Adolescents, Mental health, Primary care, Collaborative care, Middle-income countries

## Abstract

**Background:**

Child and youth mental health problems are leading causes of disability and particular problems in low- and middle-income countries where populations are young and child mental health services are in short supply. Collaborative care models that support primary care providers’ efforts to detect and treat child mental health problems offer one way to address this need. However, collaborative care for child mental health can be more complex than collaboration for adults for a number of reasons, including two-generational aspects of care, high degrees of co-morbidity, and variations in presentation across developmental stages.

**Methods:**

The study takes advantage of an existing collaborative care network in Tehran, Iran, in which general practitioners are supported by community mental health centers to care for adult mental health problems. At present, those practitioners are asked to refer children with mental health problems to the collaborating centers rather than treating them themselves. We are conducting a cluster randomized trial in which practitioners in the network will be randomized to receive training in child/youth mental health treatment or a booster training on recognition and referral. Children/youth aged 5–15 years making visits to the practitioners will be screened using the Strengths and Difficulties Questionnaire; those found positive will be followed for six months to compare outcomes between those treated by trained or control practitioners.

**Discussion:**

If the trial demonstrates superior outcomes among children treated by trained practitioners, it will support the feasibility of expanding collaborative care networks to include children.

**Trial registration:**

ClinicalTrials.gov, NCT03144739. Registered on 8 May 2017.

## WHO Clinical Trials Data Set

1. Primary Registry: ClinicalTrials.gov: NCT03144739
2. Date of Registration:
3. Secondary Identifying Numbers: none
4. Sources of support: Funded by grant R34MH106645 from the National Institute of Mental Health (US) and grant 962515 from the National Institute for Medical Research Development (Iran). The funders had no role in the preparation, review, or approval of this manuscript, or the decision to submit the manuscript for publication.
5. Primary sponsor: Johns Hopkins University, Baltimore, MD USA
6. Secondary sponsor: Tehran University of Medical Sciences, Tehran, Iran
7. Contact for public inquiries:
  Vandad Sharifi, MD
  Department of Psychiatry
  Tehran University of Medical Sciences, Roozbeh Hospital
  South Kargar Ave.
  Tehran 13334 Iran
vsharifi@tums.ac.ir
  +98 21 5541 2222
8. Contact for scientific inquiries (PI)
  Larry Wissow, MD MPH
  Division of Child and Adolescent Psychiatry
  University of Washington School of Medicine
  4800 Sand Point Way NE
  M/S OA.5.154
  PO Box 5371
  Seattle, WA 98145-5005 USA
lwissow@uw.edu
  +1 206 987 1837
9. Public title: Training GPs to care for children’s mental health problems
10. Collaborative Child Mental Healthcare in Low-Resource Settings
11. Country of recruitment: Iran
12. Health conditions studied: children’s behavioral and emotional problems
13. Intervention: Training for GPs already taking part in a collaborative care network. Training addresses detection and management of common child and youth emotional and behavioral problems. Control group GPs receive brief training in detection and referral of children to a collaborating mental health center.
14. Key inclusion criteria:
  - Participating GPs must be part of the Tehran University Collaborative Care Program
  - Children and youth must be attending the practice of a participating GP and be between the ages of 5 and 15 years old.
15. Study type: Cluster randomized clinical trial.
16. Date of first enrollment: May 2018.
17. Sample size: 355 children/youth. 234 recruited to date.
18. Recruitment status: currently recruiting
19. Primary outcome: reduction in total problems score on Strengths and Difficulties Questionnaire
20. Key secondary outcome: reduction in functional problems score on Strengths and Difficulties Questionnaire
21. Ethics review: Approved, April 27, 2018 Johns Hopkins University IRB and Tehran University of Medical Science and Health Treatment Services, Research Directorate Deputy, August 22, 2016,.
22. Date of study completion: not yet known
23. Summary of results: pending
24. Data sharing planned via the National Database for Clinical Trials Related to Mental Illness.

## Background and rationale

### Unmet need for child and youth mental health services

Worldwide, 10–20% of children and youth develop mental health problems [1]. Commonly occurring problems such as anxiety and depression can potentially be prevented or ameliorated through intervention in childhood and adolescence [2, 3] but frequently go untreated for years or decades [4, 5] causing a major burden of disability, especially in countries with predominantly young populations [6].

Primary medical care can play an important role in plans to increase access to care for children’s mental health problems [7, 8]. Primary care is widely available, allowing simultaneous interaction with children and their parents. It treats mental health in the context of medical and developmental concerns and reduces the stigma associated with visiting identifiable mental health facilities. However, to date, nearly all adult and child models for integration of mental health with primary care have targeted a narrow range of conditions at diagnostic levels and relied on additional co-located personnel. While this has demonstrated the feasibility of integration on a limited scale, it stops short of providing a readily disseminated model for broadly addressing child mental health problems, which are often subthreshold, highly co-morbid, and can involve simultaneous treatment of parents [9]. Countries across the spectrum of income and resources need integration models that address the range, complexity, and local idioms of child mental health problems, and that can be practically and effectively delivered by primary care providers themselves [1, 10].

### Models for integrated mental healthcare for children and youth

Elaborations on Wagner’s Chronic Care Model (CCM) [11] have been the basis of most programs to integrate mental health with adult and pediatric primary care. Several chronic care model-based interventions with positive outcomes have been reported for adult depression; three positive trials have been reported for adolescent depression and one for disruptive behaviors among younger children, all using additional co-located personnel [12–15]. The first step in adapting the chronic care model for a wider range of child/youth mental health problems and for use by primary care providers themselves involves developing a core set of engagement and self-management interventions: universal and problem-specific interventions that are evidence-informed; suit the patients and their problems in any given community; and are feasible in general medical settings [16]. These adapted treatments can come from several sources. First, the “common factors” literature from psychotherapy emphasizes how patient–provider interactions influence outcomes across diverse therapies and inform the CCM’s engagement and coaching strategies [17]. Studies in low- and middle-income countries also find that patient–provider interactions impact outcomes in ways similar to what is observed in higher resource countries [18]. Second, the “common elements” of evidence-based child mental health treatments suggest interventions that can be matched to child and parent concerns and delivered before completion of a formal diagnostic process [19, 20]. The reviews and expert panels conducted for the World Health Organization’s (WHO) Mental Health Gap Action Plan (mhGAP) outline interventions, suitable for delivery in general medical care, to detect and manage developmental disabilities, behavior problems, and depression [7]. Third, studies of “single session” psychotherapy (and other brief intervention models among adults) demonstrate the effectiveness of providing treatment in brief pulses across extended periods, similar to patterns of medical primary care [21]. Fourth, “stepped care” models for treating adult depression suggest that generalists can provide first-contact mental health treatment if they reliably follow patients to ascertain need for further diagnosis or intervention [22]. In a study in the United States, we found that training community-based primary care providers in “common factors” skills impacts children’s outcomes [16, 17]. We also have preliminary evidence that this approach to integration can be successfully used for care of adults with HIV in Ethiopia [23, 24].

### Context of the trial

In 1988, the Iranian Ministry of Health and Medical Education (MOH) set a goal of involving rural and urban primary care centers in the detection and treatment of both common and severe mental disorders. The program succeeded for adult services in rural areas [25], but its reliance on community health workers (CHWs) failed in urban areas where populations were more mobile and less close-knit [26]. In response, the MOH financed a pilot urban mental health collaborative care program, the Tehran University Collaborative Care Program (TUCCP), replacing CHWs with general practitioners (GPs) as the first line of services and point of access to specialists [27–30]. TUCCP spans public and private primary care sites linking > 60 urban GPs with community mental health centers (CMHCs) for consultation, referral, and ongoing support of GP skills. The CMHCs are staffed by a psychiatrist, one or two masters-level clinical psychologists, and a receptionist. For adults, the model promotes task-shifting care for common mental disorders to the GPs and stepped care via referral for individuals with more serious mental disorders. However, for children and youth, the current model calls for GPs to function only as gateways, referring all levels of problems to a CMHC.

TUCCP fully operationalizes the chronic care model and includes features advocated for US medical homes and accountable care organizations [31]. Its main components are: (1) an initial three-day training for GPs followed by quarterly boosters and ongoing interaction between CMHCs and GPs; (2) informal mental health consultation to GPs about specific patients, including facilitating referrals to CMHCs (who may take over care or return the patient to the GP); (3) a case manager role for the GPs’ receptionists, who follow patients by phone to check on their status, re-enforce interventions, and remind them of appointments; (4) a health information system for ongoing case tracking and quality assurance; and (5) payment of top-up fees to the GPs (like care management fees for US-certified medical homes), contingent on documented follow-up and adequate treatment. When a GP identifies a patient with a mental health problem, the GP or the receptionist enters demographic and diagnostic information and the treatment plan into the information system. A psychologist and psychiatrist from the CMHC regularly monitor the information system and call the GP offices to monitor follow-up and give feedback on treatment plans. Every three months, TUCCP staff visit offices to audit a sample of charts and discuss management. TUCCP staff also regularly call a 5% sample of enrolled patients to assess satisfaction with the services from GPs and their receptionists.

Presently, the TUCCP focuses on collaborative care for adults. Physicians participating in the TUCCP are advised to refer identified children/youth with mental health problems to CMHCs for further care; however, only 192 child/youth patients were referred in the TUCCP’s first 30 months, substantially fewer than had been expected based on the volume of children seen by GPs. Of these children, only 67% completed their referral. GPs believed that a majority of parents would welcome alternatives to referral to CMHCs. They said that parents declined CMHC referrals because of distance, stigma, and concern that specialists would propose medication rather than psychotherapy. TUCCP records suggested that > 75% of patients referred by the GP’s had conditions (ADHD and anxiety) that could potentially have been treated in primary care had that been permitted under TUCCP protocol.

## Objectives

The study is designed to collect both effectiveness and implementation outcomes with a goal of understanding mechanisms by which both the implementation and effectiveness outcomes are achieved [32]. In Curran et al.’s typology of hybrid studies, this would be designated as “Type 1” – the primary question is whether the intervention will work in this setting, and the secondary question concerns potential barriers and facilitators to implementation. Criteria for Type 1 studies include “strong face validity” for the intervention’s fit to a new setting, strong evidence from other settings supporting applicability, and minimal risk either of displacing existing effective care.

The specific goal of this study is to conduct a hybrid effectiveness-implementation trial with TUCCP GPs focusing on the following questions:

1. Whether care delivered by primary care providers results in improved child and parent mental health outcomes?
2. Exploring the mechanisms by which primary care achieves those outcomes. Which conditions are more likely to be identified and treated by GPs? Which treatments have the greatest uptake by parents and youth?
3. Does offering treatment in primary care result in a larger proportion of children with mental health problems receiving treatment?
4. Whether a coordinated program of training, ongoing coaching, and collaborative care results in uptake by primary care providers as evidenced by treatment provided in primary care and participation in collaborative care through consultation and referral?

### Hypothesis

Children/youth with emotional and/or behavioral problems, as identified by parent reports (the Strengths and Difficulties Questionnaire [SDQ]) [33] will have reduced symptoms and improved functioning at a six-month follow-up point when they are treated by a trained TUCCP GP compared to children receiving usual care (treatment by a provider who can offer only referral to a CMHC).

## Methods

### Trial design

The study has a cluster randomized, staggered start, parallel design in which the recruited GPs will be randomly assigned to one of three training waves, and within these waves to either intervention or control arms. Children/youth and one of their parents will then be in the intervention or control group depending on their GP’s assignment.

### Setting

Iran is classified by the World Bank as a middle-income country (per capita annual income in the range of US$1026–12,475 in 2011 dollars) [34]. Tehran is the capital of Iran and a city of about nine million people centering a metropolitan area of about 15 million people. The CMHCs involved in the TUCCP are located in southern districts of the city, areas with relatively high literacy rates but paradoxically higher rates of unemployment and greater housing density [35]. Most of the population is of Fars or Azari background, but these districts also house refugees from several neighboring countries.

Care in Iran’s medical system is financed by a mixture of public and private insurance. In recent years the government has implemented plans to reduce the number of uninsured and to reduce the proportion of out of pocket health expenditures by about 50% [36]. In the TUCCP, patients pay for GP services out-of-pocket or through health insurance but receive CMHC services free of charge. Most of the GPs who participate in the TUCCP are in solo practice in offices where their additional staff may be limited to a receptionist. The CMHCs are staffed by a general psychiatrist, one or two masters-level psychologists, and a receptionist.

### Participants

#### Children/youth and their parents

The main participants in the trial are children/youth aged 5–15 years (male and female) and one of their parents or guardians (whoever accompanies the child/youth to their GP visit). These children will generally be coming to the GP for a non-emergent medical, emotional, or behavioral concern. Some children may have a chronic medical or mental health condition, but we will not recruit any child/youth who reports being in pain or who appears to be acutely medically ill. Children/youth who are actively treated at the CMHC at the time of recruiting will be excluded, as will any who do not speak Farsi. We plan to recruit approximately 1500 children/youth and their parents/guardians (screening step) and follow about 350 of each (children who screen positive for an emotional or behavioral problem on the SDQ and their parent/guardian) by telephone at three and six months after the index visit. The number of children screened may be smaller or larger depending on the proportion screening positive.

#### GPs

We will recruit from among GPs who are already taking part in the TUCCP. All 60 GPs currently in the program will be eligible to participate but we have based power calculations on recruiting 45 participants. We will aim to enroll a minimum of 48 and a maximum of 60 to allow for attrition before the start of training.

### Recruitment and screening

GPs will be approached by TUCCP staff with whom they already interact regularly. If they express interest in participating in the study, one of the Iranian investigators will contact them, provide more information, and obtain written consent. GPs would be excluded only if they reported caring for none or only a negligible number of children.

#### Children/youth and their parents

Once GPs are enrolled, randomized to a training wave and intervention or control arm, and trained, we will recruit consecutive eligible patients from their offices. Thus, children and their parents will automatically be in the group (intervention or control) to which their GP has been assigned. As noted below, the consent process will disclose that the GP is participating in the study but not say to which group the GP has been assigned. Families will be told, however, that their GP participates in the collaborative care program and that they are free to request a referral to a CMHC.

With the help of receptionists, families will be approached by a research assistant when they arrive at a participating GP’s office and asked about their child’s age and their interest in participating in the study. Families will not be approached if the child appears to be acutely medically ill or in pain. If the family is interested, they will be taken to a private space to discuss the study and to consider consent/assent. After confirming eligibility, parents will be asked to consent to baseline assessment, access to their child’s TUCCP record if one is created, to receive follow-up calls at three and six months if their child scores “positive” on the SDQ, and to participate in a follow-up interview.

Consenting parents will be asked to complete the parent report version of the SDQ that corresponds to the age of their child (see below). SDQ results will not be shared with the GP and summary scores will not be discussed with parents. Parents and children/youth, however, will be free to discuss the SDQ with the GP or anyone else; and research assistants administering the SDQ and other baseline instruments will encourage parents to discuss any concerns they have with their GP.

We will attempt to complete the initial SDQ at the time of the family’s GP visit, but if this is not possible, for reasons of time or lack of privacy, the SDQ will be completed by telephone soon after the index visit. While literacy rates in the study area are relatively high, assistants will be prepared to read the SDQ items to parents if required.

### Trial follow-up

Those children/youth who screen positive on the SDQ will be considered to have entered the longitudinal phase of the trial and will be followed by telephone with re-assessment at three and six months after screening (regardless of whether or not the GP has considered them to have a mental health problem and entered them in the TUCCP information system).

## Randomization and treatment allocation

In this cluster randomized trial, the unit of randomization is the GP. Randomization using the Stata procedure “ralloc” (StataCorp, College Station, TX, USA) will assign the GPs to one of three training waves and to the intervention or control arms. The Iranian team will provide the US team with a list of study numbers corresponding to enrolled GPs. The US team will provide the Iranian team with a list of allocations balanced across waves, public/private practice site, and overall volume of patients seen at the practice. In the event that a GP is unable to attend training for her or his assigned wave, the Iranian team will exchange that GP for a GP with the same treatment/control allocation from another wave. This means that some waves may become slightly imbalanced with regard to GP practice characteristics but balance across the study will be preserved. If GPs who initially indicated interest say that they cannot attend any of the scheduled trainings, new GPs with the same characteristics (public/private, large/small practice) may be substituted if available. Children/youth and their parents will be clustered within GPs and thus have the same control/intervention status as their GP.

## Masking

Research assistants making follow-up calls will not be blind to the child’s control/intervention status because assistants who recruit families (and collect information from GPs about their initial assessments) will, when possible, make the three- and six-month follow-up calls to the same families they have recruited. However, the assistants will have no knowledge of any data about the child from the TUCCP database and will administer the SDQ before other instruments that might reveal information about treatment received after the visit at which the child was enrolled. We believe that the families’ familiarity with the research assistant, coming from an in-person contact at the time of consenting, will promote follow-up and that this advantage balances concern about biased outcome assessment.

Similarly, it is not possible for GPs to be masked. Consented parents and youth who receive care for a mental health issue during the study are likely to discern whether their GP is in the intervention or control arm of the study and might even question their GPs about their assignment to the study arms. The GPs will be encouraged to share this information with the families if they wish so.

## Interventions

### Intervention arm

Training content (Table 1) is derived from past work in training of primary care mental health skills [17], the WHO’s Mental Health Gap (mhGAP) intervention guide [37], and from information gained in the formative phase of the project. The intervention training is designed to help GPs identify child and youth emotional and behavioral problems, engage families in care, provide first-line interventions, and refer to the CMHC based on family need or desire. The training takes a trans-diagnostic approach to mental healthcare, based on: (1) universally applicable communication skills; (2) broadly applicable techniques including problem solving, emotion regulation, and help with parent–child interactions; and (3) use of problem-specific brief treatments drawn from mhGAP. Work in the formative phase led to the addition of substance problems and special attention to the needs of immigrant families. The Iranian investigators compiled these topics into a training manual (trainer and trainee versions) accompanied by scenarios for role plays with standardized patients. The training was pilot tested with a group of 18 GPs participating in a collaborative care network in a different city.

**Table 1:** Content areas for child/youth mental health training

Topic	Brief interventions
Patient–provider interaction	Engaging patients in conversation, attentive listening, eliciting an agenda, empathy, giving advice, decision support, interacting with child and parent
Children with problematic behavior	Assessing possible causes (anxiety, depression, trauma, developmental delay or school problems, attention problems), general parenting advice, help with schoolwork, psychoeducation for families about children with delays, linking to community resources for children with developmental delays, appropriate medication use
Depression (child and/or parent)	Psychoeducation, behavioral activation, exploration of contributing stresses, exploration of suicidal ideation, safety for suicidal patients, medications for older adolescents and parents
Anxiety	Psychoeducation, active coping (role models, supported exposure, relaxation), support and response to maladaptive cognitions following trauma, exploration of contributing stresses, indications for medication
Substance use	Recognition of harmful use of tobacco, alcohol, and inhalants. Brief counseling regarding cessation
Intellectual disability and learning disorders	Methods of detection, behavioral and pharmacological management of associated behavior and mood problems
Urgent issues	Thought problems, suicidality, intentional harm to children or risk of harm to others
Parenting and typical developmental issues	[in addition to behavioral topics above] sleep, toileting [including enuresis and encopresis], masturbation

Training for the intervention arm GPs will involve a 2.5-day in-person session with role plays and case discussions followed by an in-person booster session two months later. The booster will focus on family engagement, psychoeducation, and trouble-shooting problems that the GPs encounter in providing child mental healthcare. It will use an active format, including repeating three common scenarios from the initial training (demonstrations by trainers and standardized patients and practice by the attendees), adding discussion of actual cases the GPs have encountered, and reviewing study procedures.

### Control arm

Control GPs will receive a one-day child/youth mental health refresher course that will focus on identification of child and youth emotional and behavioral problems. The material will expand on past TUCCP trainings and cover only problem recognition and discussion with families about treatment options available through the CMHCs. As with the intervention arm training, control training will include role plays with standardized patients. The scenarios used will mirror those used in the intervention arm but will be focused on detection and referral rather than management.

### Support/interventions common to both arms

GPs will receive the equivalent of about $200 as compensation for their training time and for completing study instruments. As part of their participation in the collaborative care program, they will also receive incentive payments for successfully managed cases. As currently defined by the TUCCP, successful management for patients treated by the GP is defined as: (1) monthly visits with the GP for the first three months after treatment initiation or substantial change in treatment; and (2) follow-up visits at no more than three-month intervals for patients who are stable. The amount received varies according to a quality rating assigned by the TUCCP. GPs do not receive additional payments for patients they refer.

Following training, all GPs will have continuous support from mental health center staff who will provide them with feedback about registered cases and who are available for informal telephone consultation. Staff working with control GPs will follow current TUCCP protocols for child/youth patients; they will support GP problem recognition and referral for assessment and care. Staff working with intervention GPs will be trained to: (1) help GPs assess whether a given patient’s problems are suitable for treatment in the primary care setting; and (2) if so, help the GPs apply treatments discussed in the training.

### Fidelity

The competence of GPs to deliver child/youth mental health treatments, or to refer for services, will be assessed in two ways. First, as mentioned above, intervention GPs will participate in role plays with standardized patients as part of their initial training. Structured observations of these assessments will be used to measure uptake of the training received. Second, TUCCP records and structured supervision reports from TUCCP staff will be used to determine the extent to which participating GPs are providing care beyond referral and the degree of additional support required to assure that interventions provided by the GPs are consistent with the intervention training. Comparison of TUCCP records with study data will document the proportion of SDQ-positive children identified as having a mental health problem and offered some form of treatment.

## Outcomes

The trial outcomes are summarized in Table 2.

**Table 2:** Summary of outcome measures for the TUCCP child/youth mental health collaborative care trial

Outcome	Measure	Timing
Primary effectiveness outcome		
1. Total child/youth behavioral/emotional problems score	SDQ	Baseline, three, and six months
Secondary effectiveness outcomes		
1. Child/youth function score	SDQ	Baseline, three, and six months
2. Parent mental health status	GHQ	Baseline, three, and six months
3. Parent functional status	EuroQol EQ-5D-5 L	Baseline and six months
4. Satisfaction with GP care	Index visit exit questionnaire	Baseline
	In-depth interviews (sample)	Three and six months
5. Use of mental health services	Community services report	Baseline and six months
	TUCCP utilization data	Six months
Implementation outcomes		
1. GP mental health skill uptake	Standardized patient role plays	Baseline and three months
2. GP recognition of child/youth mental health problems	GP exit form	Baseline
	GP registration of patient in the TUCCP system	Baseline
2. GP treatment or referral for major mental health problem categories	TUCCP operational data	Six months
3. GP’s confidence in treating mental health problems	Confidence scales	Immediately after training
4. GP’s attitudes toward mental healthcare	Physician Belief Scale	Immediately after training
Potential moderating variables		
1. Parent and youth demographic characteristics	Self-report of age, gender, education, economic status, ethnicity	Baseline
2. Parent and youth relationship to GP office	Self-report of number of prior visits, distance from home to office	Baseline
3. GP characteristics	Training, prior activity in TUCCP, length of time in practice at current location, overall practice volume	Baseline

### Primary outcome

The primary outcome is the total problems score on the SDQ. The SDQ is widely used internationally. It is a brief (25-item) tool with the advantage over other brief tools that it includes positive (pro-social) items and includes items assessing function as well as symptoms. The Farsi parent report SDQ for younger children has been validated against the Farsi Child Behavior Checklist [38] and the K-SADS present and lifetime version [39]. The SDQ score distribution in the Iranian sample was similar to that reported in other countries. In a separate study, the parent report SDQ was tested for children/youth aged 3–18 years [40] and found to have good internal reliability. In other countries, the SDQ has shown good sensitivity to change over time [41]. In a pilot study, the Iranian investigators of the current trial found that telephone and in-person results for the parent-reported SDQ were highly correlated.

### Secondary outcomes

Child/youth functional status will be measured with the functional assessment items from the SDQ. Especially among younger children, large discrepancies can exist between the presence of symptoms, which may be prominent, and functioning, which may seem minimally impaired compared to the severity of symptoms [42].

Parents’ mental health status will be measured both as a secondary outcome and as a potential moderator or mediator of child mental health outcomes [43, 44]. Parental emotional status will be measured at baseline, and at the three- and six-month follow-ups with the 28-item self-report General Health Questionnaire (GHQ) [45]. The GHQ can be used categorically with good sensitivity and specificity for psychiatric disorder and as a continuous measure of distress. Its questions are framed in terms of recent changes in mood, which make it suitable for use in longitudinal studies, and it has been previously used in in Iran [46]. Parents’ overall functional status will be measured with the EuroQol EQ-5D-5 L, a brief (six-item) instrument that measures five areas of functioning (mobility, self-care, usual activities, pain/discomfort, anxiety/depression), each at five levels [47]. It will offer the opportunity to understand the impact of parental disability which may or may not include mental health concerns disclosed on the GHQ. The EQ-5D-5 L can be administered by telephone and is sensitive to a wide range of chronic conditions including mood and chronic pain problems in the general populations [48]. Unlike many instruments, the EQ-5D-5 L is scored by comparing the pattern of responses across the five areas of functioning to population-based norms. A Farsi version of the EQ-5D-5 L with scoring norms has been developed for the Iranian population [49].

Parent satisfaction with care will be assessed by first asking parents if the visit involved discussion of a mental health problem and, if so, whether the GP suggested a plan for treatment [50]. Subsequently, we will ask about satisfaction using questions initially developed by Zastowny et al. [51] based on Hulka’s [52] conceptualization of satisfaction with providers’ interpersonal skills and technical competency. Two questions ask about overall satisfaction with the visit, three reflect satisfaction with informativeness (was the doctor’s information helpful, did the doctor clearly explain what you should do, answer all your questions) and four reflect partnership (did the doctor encourage you to talk about your worries, ask for your opinion about treatment, spend enough time with you, treat you with respect). In past work [53], we found that while the overall satisfaction and informativeness items were skewed to higher ratings the partnership items are sensitive to differences in provider interaction style, which is targeted by one of the components of the intervention training.

From the TUCCP data system, we will be able to determine treatment received by participating children from either the GP or the CMHC. At baseline and at the six-month follow-up point, parents will be asked to complete a questionnaire asking about the use of mental health or counseling services that they may have received outside the TUCCP (from schools, clergy, or other mental health providers).

### Implementation outcomes

We will examine several measures that, in addition to the fidelity measures described above, will help to understand the mechanisms by which the intervention arm training may produce the child/youth and parent-level outcomes. These include differences between the intervention and control arms in the proportion of children treated by GPs rather than being referred to the CMHC, increases in GPs’ confidence in their ability to manage child/youth mental health problems, and changes in attitudes toward taking on mental healthcare of children in general. To measure attitudes, we will use the burden subscale of the Physicians’ Belief Scale [54], which has been found to be inversely correlated with identification of psychosocial issues [55]. The TUCCP data system will also be able to provide information on quality of care provided by GPs (routine ratings related to top-up payments as well as subjective ratings from supervision reports).

### Baseline characteristics

We will measure several baseline factors to characterize our sample, be able to adjust analyses in the case of unequal distribution between the intervention and control groups, and for further analyses to explore subgroups for which the intervention may be more or less effective. These include, for children/youth, family demographics (income, parental education, ethnicity, distance from treatment sites), age, and gender. While we do not expect that age and gender will vary between children/youth in the two study groups, it is possible that there will be differences in age or gender of children who actually receive treatment. We will also examine GP factors that could be related to implementation outcomes, including training, prior activity in TUCCP, length of time in practice at current location, and proportion of children in the overall practice volume.

## Sample size calculation

We have powered the study to find a difference of about 2.5 points on the SDQ’s total problems scale (the sum of all items on the emotion, conduct, hyperactivity, and peer problems subscales [33]) when comparing the intervention with control group at the six-month observation point. This difference corresponds to an effect size of about d = 0.34. It is the size of change measured using the SDQ in a study of a parenting intervention with Iranian mothers [56] and is similar to effect sizes of trials in high-income countries [17, 57]. An estimated standard deviation of 7.2 was also drawn from these sources. Correlations between baseline and outcome measures and between outcome measures were estimated at 0.37 and 0.55, respectively, also based on prior data from the US [17].

The required patient sample size was estimated in four stages. We first used the “change” option in the “sampsi” routine in Stata Release 12 (StataCorp, College Station, TX, USA) to obtain a sample size disregarding clustering among GPs. The “change” option computes a t test on the difference in change scores (outcome minus baseline) between the intervention and control groups. The variance is adjusted for correlations between the outcome measures and between the outcome and baseline measures. With alpha = 0.05 and power of 0.8 the routine yielded a sample size of 135 per study arm (270 total).

In the cluster randomized studies, sample size is dependent on the extent to which outcomes are correlated within clusters. A review of adult primary care studies from high-income countries found intraclass correlation coefficients for depression outcomes in the range of 0.016–0.035; the range for the SF-36 mental health measure was 0.009–0.033 with an overall median of 0.01 [58]. Given that we plan to enroll 45 GPs, we would have clusters of six patients per GP (6×45 = 270) yielding a design effect of 1 + (6–1)×0.01 = 1.05, which would increase the sample size needed at outcome to about 284. Assuming a worst-case of 20% loss of patients to follow-up would require enrolling 355 patients.

There are varying estimates of the prevalence of high/abnormal SDQ scores (≥ 17). We found 24% to be ≥ 17 in a study of children aged 6–16 years in primary care in the US [16]; Alavi et al. [59] found a prevalence of 26% using this scoring of the SDQ in a population-based sample of children aged 6–11 years in Tehran. Using an estimate of 24%, we then need to screen about 1479 children/youth to obtain 355.

## Data management and analysis

### Data management

Incoming data from GP and patient instruments will be logged and examined for completeness and ambiguous entries. Queries will be sent to the research assistants to examine possibilities for re-contacting participants. Research assistants will use a messaging application to report on their daily progress with recruiting or follow-up and to be able to clarify or report any study issues immediately. Data collected on paper will be double entered. For each research assistant, a 5% sample of the participant forms that they collect will be checked by having a different member of the study team call the participant to verify key items. Supervisors of research assistants will make random visits to GP offices while research assistants are present to check on recruitment and data collection processes.

A trial data center in Tehran will be responsible for: (1) scoring baseline SDQ forms to determine which children/youth are eligible for the trial; (2) maintaining files that link GPs to intervention status and children/youth to follow-up contact information; (3) merging data collected directly from GPs and patients with data extracted from the TUCCP data system; and (4) creating and maintaining unlinked merged data files. Duplicate merged unlinked files will be sent to the US study site for analyses, distribution to the Data Safety and Monitoring Board, and archiving. A final dataset will be generated and deposited with the National Database for Clinical Trials Related to Mental Illness [60].

### Data analysis approach

Analysis will follow a predetermined plan and take place under the direction of the trial statistician. Analyses will be masked to study arm until data collection is finalized. Exploratory analyses will confirm expected distributions and the prevalence and patterns of missing data. We will use appropriate methods for missing or censored data, such as multiple imputation or full information maximum likelihood [61].

The outcomes in each set of analyses will first be explored with simple bivariate statistics. Multilevel models with random effects will model patient outcomes, which are clustered within GPs [62]. The basic approach has the following form: if the study ultimately has *I* clusters (i = 1:I), 3 time points (j = 1:3), and *N* patients per cluster (k = 1:N), individual patient outcomes are modeled as *Y*_*ijk*_ = μ_ij_ *+ γC*_*ijk*_ *+ e*_*ijk*_ where *e*_*ijk*_ has a ^*iid*^ *N(0,σ*^*2*^_*e*_*)* distribution. Individual covariates included at this level (denoted by C) will include child age, gender, and initial SDQ symptom and functioning scores. The mean outcome for cluster i at time j can be expressed as: *μ*_*ij*_ *= μ + α*_*i*_ *+ βZ*_*ij*_ *+ X*_*ij*_*θ,* where *α*_*i*_ is a random effect for the cluster, *Z*_*ij*_ are the cluster-level covariates (e.g., public or private GP practice, practice size, the three staggered training waves to which the GP belongs), and *X*_*ij*_ = 0 if the cluster is a control practice or = 1 if it is an intervention practice.

Interaction terms between intervention status and a hypothesized cluster-level moderator (e.g. GP’s public or private practice, practice size, training wave) will be used for exploratory analyses of effect heterogeneity.

Analysis will be by intent-to-treat based on the intervention status of the GP. However, intent-to-treat analysis does not take into account the level of intervention actually received by individual patients. Therefore, the intent-to-treat data will be presented along with data describing, for each arm: (1) the proportion of patients receiving any form of collaborative mental health treatment; (2) the proportion of patients who do receive mental health treatment who receive it solely from a CMHC; and (3) the proportion of patients whose GP left the collaborative during the course of the study. Only item (3) will be considered a protocol deviation, since per the protocol, GPs may opt to treat or refer any patient. In addition, the impact of implementation outcomes such as GP changes in attitude and confidence (“post-treatment mediator” variables) will be examined using causal mediation methods [63].

The study team plans to develop papers and presentations related to the study’s objectives for appropriate national and international journals and meetings. The team is committed to developing opportunities for early career investigators involved in the study to take leadership roles in preparing reports.

### Interim analysis

**Fig. 1:**
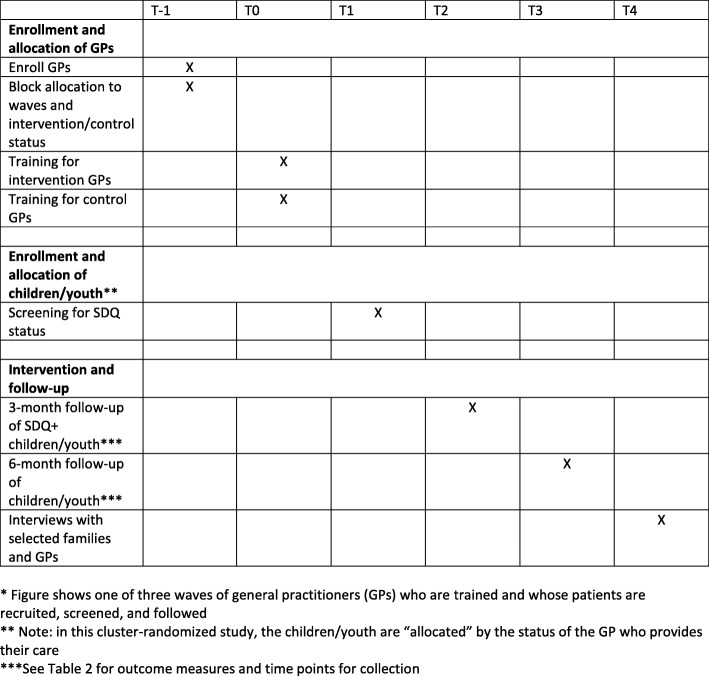
Simplified* flow of study procedures. *Figure shows one of three waves of general practitioners (GPs) who are trained and whose patients are recruited, screened, and followed. **Note: in this cluster randomized study, the children/youth are “allocated” by the status of the GP who provides their care. ***See Table 2 for outcome measures and time points for collection

An interim analysis will be conducted when patients recruited from the first wave of GP practices have reached the six-month follow-up point (Fig. 1). At this point, patients from the second wave of GP practices will have been enrolled and training will be taking place for GPs in the third wave. The US study team will use unlabeled data to assess the primary outcome, adverse events, and TUCCP referral or treatment data by arm and present the results to the DSMB for their assessment. It is unlikely that major differences will be observed in the primary outcome at this point, but there could be differences in adverse events or in the proportion of children receiving care that the DSMB might question.

## Ethical considerations

Ethical approval for the trial has been obtained from the Committee for Ethics in Research of the Tehran University of Medical Science and Health Treatment Services (Project 32,376) and from the Institutional Review Board of the Johns Hopkins Medical Institutions (IRB00166709). These IRBs will receive reports of any adverse events and will approve protocol changes as necessary. The study’s NIMH project officer will receive reports from DSMB meetings, annual progress reports, and other updates as necessary or requested.

### Events that would preclude the participant from continuing in the study (other than their own withdrawal)

For GPs, it is possible that the TUCCP internal monitoring system could decide that an intervention GP was not able to properly handle child/youth mental health cases. The system might request that the GP go back to the baseline situation of referring all child/youth issues directly to the CMHC for treatment. This would not be a reason for the GP to leave the study. The GP would still be considered an intervention arm GP as per the intent-to-treat design. TUCCP has a monitoring plan to review practices every three months and to decide if they can continue in the collaborative. The plan includes monitoring routine electronic clinical data/patient records, monthly quality assurance face-to-face meetings with the GPs’ receptionists, and face-to-face meetings with each GP every three months. It is possible that a GP would be asked to leave the TUCCP, in which case they would be considered to have left the study.

For children, inability to contact an appropriate guardian to obtain follow-up information would be reason for withdrawal.

### Privacy for children/youth and parents

The information collected in the course of the study does not go beyond what could be collected in the course of routine general medical care that follows international guidelines for inquiry about psychosocial as well as physical health [6]. However, unwanted disclosure of this information to third parties could be socially damaging or traumatic to the children/youth or their families. Other than the loss of study data, the main threats to privacy are unwanted disclosure during attempts to follow-up on SDQ+ children/youth. We will try to minimize this risk by training staff regarding confidentiality and security of data.

The SDQ does not ask about self-harm nor victimization that could prompt a need for involuntary breach of confidentiality. The GHQ (for adults only) does have questions about thoughts of self-harm but no questions about exposure to violence. None of the child/youth or adult instruments used in this study include questions about substance use, sexual orientation/activity, or other behaviors that might be considered illegal or especially stigmatized in Iran. Both the SDQ and GHQ have been previously used and found to be culturally acceptable in studies in Iran.

It is possible that some youth participants could disclose information to their GPs that they might not want to be disclosed to their parents. In Iran, it is common for teens or even adults to come for visits with a family member who remains present in the examination room during the visit. However, GPs can choose to ask family members to step out to afford the patient some privacy. On the other hand, it is possible that responding to the GHQ could prompt parents to want to discuss issues with the GP that they would also prefer to discuss in confidence and they may ask their child to leave the room.

GP training for both arms of the study will include discussion about the importance of asking about substance use and exposure to intimate partner violence, but this would take place as part of the clinical encounter and be at the GPs discretion. GPs will be trained in the appropriate procedures to follow should a concern for child maltreatment arise during a visit. *Currently there is no legal obligation for GPs or study personnel to report suspected child maltreatment to authorities in Iran. Reporting obligations only apply to hospital staff for children who have been hospitalized.* TUCCP consultants are capable of helping GPs review options for responding to concerns about maltreatment and other forms of family violence.

### Privacy for GPs

GPs could experience risk to their careers if they make comments during pre- or post-study activities that could be perceived as critical of colleagues, authorities, or other professionals from whom they themselves may seek care. GP performance with standardized patients could subject their skills and knowledge to a level of scrutiny that they would not ordinarily experience in the course of their regular practice. All GP data will be treated confidentially.

### Coercion to consent and involuntary participation for children/youth and parents

Among the participants to be recruited, the children/youth by definition fall into the category of a potentially vulnerable population. However, parents may also be vulnerable for reasons of poverty, low literacy, or mental health problems of their own. We will make sure that the parents and children clearly understand that whether they choose to participate in the study will have no impact on the care they receive from the GP. We will accomplish this by including clear language in the consent and assent and by employing research staff that are not employees of the general practice.

### Economic burdens for families

Follow-up care in CMHCs under the TUCCP is free to families, but GPs charge for follow-up care. On the other hand, families may incur considerable opportunity and travel costs to attend CMHC visits because the CMHCs, in contrast to GP offices, are often not located in the neighborhoods where the patients live. Thus, we believe that out-of-pocket costs for families would be similar regardless of the treatment option available to them. Parents in both arms of the study will be free to request referral to a CMHC for themselves or for their child.

### Cluster design

This study uses a form of cluster randomized design in which patients cannot chose to be randomized or not because randomization has taken place at the GP level. For example, a child or youth could decline to be in the study (not be screened and not participate in the evaluation of the GP training) but could still be offered mental health treatment by a GP if their GP was in the intervention arm [64]. Cluster randomized trials in which providers or sites are the unit of randomization have been criticized because they do not allow patients a choice of treatment. In these trials, consent serves only as notification that they may be receiving a different treatment and allows them to agree to participate in an evaluation. However, intervention GPs in this study will not be obliged to treat patients themselves (they can still make referrals to the CMHC based on their own or their patients’ preferences) and patients will be able to either request a referral to the CMHC or access other mental health services regardless of the GP’s decision.

### Coercion to consent and involuntary participation by GPs

GPs might feel obligated to participate in the new child mental health training in order to maintain their affiliation with TUCCP. We will make it clear that participation is voluntary.

### Risk of inferior treatment of children/youth and parents

Currently in the TUCCP, children that the GP believes may have a mental health problem are, by protocol, referred to a CMHC for treatment. This treatment could be superior to what a GP could offer. However, we believe that there is considerable under-identification of children with potentially treatable problems and that many children who have problems detected currently are not referred, decline referral, or do not complete referrals to a CMHC. However, there remains the risk, following GP training, that GPs will attempt to treat children who should be referred or will not realize that a child’s condition has not responded to treatment in primary care and now requires referral. We minimize this risk by specifically discussing, during the GP training, scenarios in which children need to be referred to the CMHC. In addition, the TUCCP monitors treatment and follow-up as part of its routine processes as noted above.

### Burden of additional work for GPs

GPs may face additional clinical burden if their practices detect and manage more child or parent mental health problems. As noted before, there is reason to believe that child mental health problems are under-recognized in TUCCP practices and those that are recognized presently are referred. However, GPs will receive additional compensation for caring for identified children (both usual per-visit charges plus TUCCP top-up payments for successful management).

## Discussion

Worldwide, deficiencies in the availability and quality of children’s mental health services are believed to be among the key barriers to broader child development goals, including successful transition to meaningful productive adulthood [65]. Most adult mental health problems have their onset during childhood and youth; yet treatment is often delayed for years during which time individuals experience considerable morbidity and missed opportunities. In many middle-income countries, access to general medical care is good and mental health services for adults are available, but mental healthcare for children is lacking. Capacitating primary care providers to provide child mental health services has the potential to increase the supply of these services at the community level.

Collaborative mental healthcare for adults with common mental disorders has proven to be feasible and effective, but evidence for child/youth collaborative models is scant. Experience with adult collaboration models is relevant but not sufficient to inform the design of child collaborative care. This study seeks to provide initial evidence that collaborative care is feasible and effective, as well as information about models of training and conditions for which collaborative care may be best suited.

### Trial status

Recruiting GPs for the trial began in April 2018; patient enrollment began in May 2018 and is ongoing (expected conclusion March 2019). Follow-up is expected to be concluded in October 2019.

## Data Availability

A final dataset will be generated and deposited with National Database for Clinical Trials Related to Mental Illness.
